# Supplementary figures and images for: A Novel Preclinical In Vitro 3D Model of Oral Carcinogenesis for Biomarker Discovery and Drug Testing
Source: Int J Mol Sci. 2023 Feb 17;24(4):4096. doi: 10.3390/ijms24044096 (PMC9967961; doi:10.3390/ijms24044096)

## Slide 1
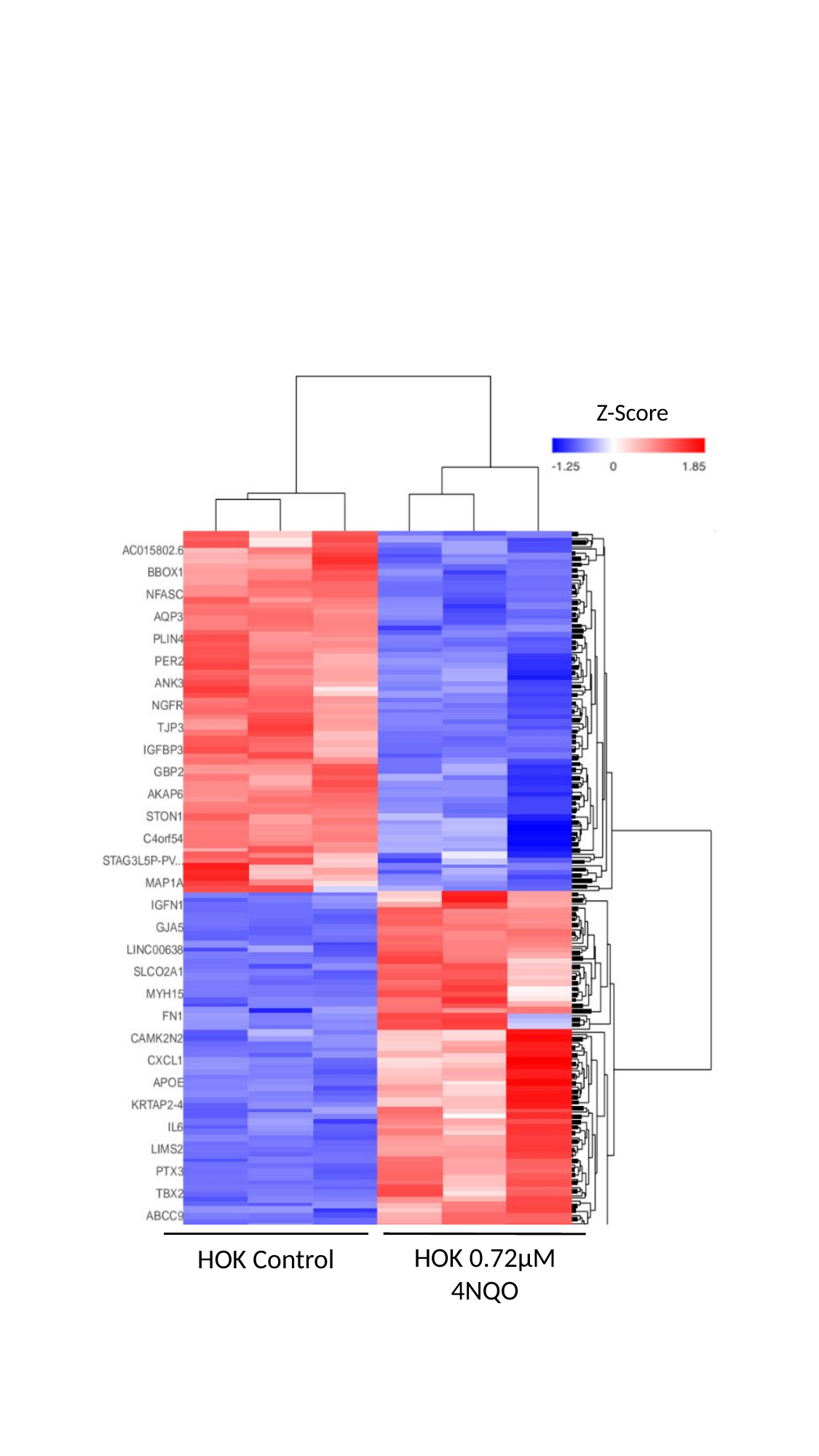

Z-Score
HOK 0.72µM
4NQO
HOK Control

## Slide 2
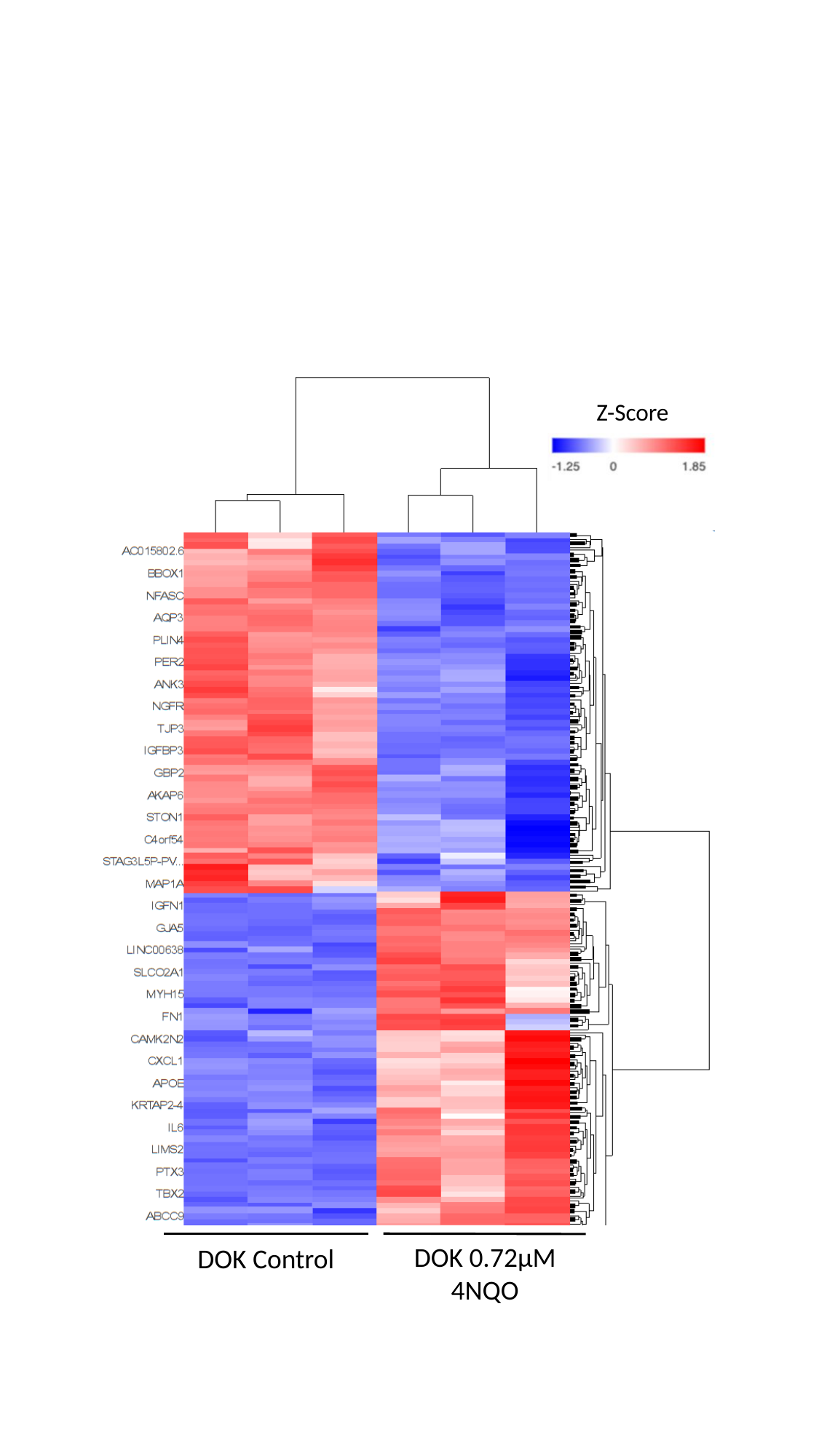

Z-Score
Z-Score
DOK 0.72µM
4NQO
DOK Control

Supplement: Supplementary file 1 [file ijms-24-04096-s001.zip › ijms-2206643-supplementary/Supplementary materials/Supplementary file S4.pptx]
